# Improving detectability of illegal fishing activities across supply chains

**DOI:** 10.1038/s44183-025-00134-5

**Published:** 2025-06-21

**Authors:** Rodrigo Oyanedel, Stefan Gelcich, E. J. Milner-Gulland, Chris Wilcox

**Affiliations:** 1 MAR FUTURA, Navidad, Chile; 2 Instituto Milenio en Socio-Ecología Costera (SECOS), Pontificia Universidad Católica de Chile, Santiago, Chile; 3 Department of Biology, University of Oxford, Oxford, UK; 4 Center of Applied Ecology and Sustainability (CAPES), Pontificia Universidad Católica de Chile, Santiago, Chile; 5 Marine Program, WildAid, San Francisco, USA; 6 Centre for Marine Sociecology, University of Tasmania, Hobart, Australia

**Keywords:** Marine biology, Sustainability

## Abstract

Improving detectability (i.e., enforcers’ capacity to detect illegal fishing activities) is vital for fisheries management, food security, and livelihoods. Identifying factors linked to higher probabilities of illegal activities and their detection across supply chains is essential for effective interventions. Using a Bayesian Hierarchical Model and a large enforcement dataset from Chile, we evaluated determinants of detectability and violation probability across supply chain actors, species, regulations, and effort predictors. Our findings reveal an overall detectability rate of 7%, varying significantly across supply-chain actors. Notably, those higher in the supply chain, such as processors and restaurants—despite receiving less enforcement effort—show higher detection rates. This study offers insights to enhance detectability and improve enforcement targeting, particularly where budgets are constrained. Our approach complements technological advancements like satellite monitoring and supports strategies to reduce illegal fishing and promote compliance, contributing to better management and sustainability of fisheries in Chile and beyond.

## Introduction

Fisheries are a crucial source of livelihood and nutritious food and can be a critical economic activity for coastal communities^1–3^. However, these benefits can be undermined by illegal activities that threaten local livelihoods, drive stock overexploitation, and impede management and conservation efforts^4–6^. A widely used tool for reducing illegal fishing activities is enforcement, which can help incentivize compliance^7–9^. However, enforcement efforts are not always effective at reducing illegal activities, or their effects cannot be properly assessed^10–12^. As such, improving the capacity of enforcers of fisheries regulations to detect illegal activities is crucial for maintaining ecologically and economically sustainable legal fish trade, which supports the nutrition of millions worldwide^2^.

Enhancing the capacity of enforcers to detect illegal activities presents a critical challenge in fisheries and natural resource management and enforcement^8,13^. Detecting clandestine behaviors is inherently difficult due to their cryptic nature, yet essential for dissecting patterns that might otherwise remain obscure^5,14^. Detectability, defined as the probability that an enforcement action detects an illegal activity, can be improved by recognizing that both illegal activities and their detection are not randomly distributed, but follow patterns and concentrate in space and time^10,15,16^. Gaining insights into the factors that determine these patterns can contribute to enhancing the detection of violations, ultimately reducing the incidence of illegal fishing activities.

A commonly collected, yet seldom used, data source to assess the detectability of illegal fishing activities is violations reports (i.e., information gathered by enforcers when they encounter an illegal fishing event while patrolling)^16,17^. However, these reports might suffer from unquantifiable biases if the only information reported is what was detected and not the type and magnitude of effort expended^18–20^. This is because enforcement can be reactive and non-random in nature (e.g., enforcers going to areas where they expect to find a violation or are used to working, instead of distributing enforcement effort in a more systematic way); therefore, enforcement data are inherently biased^8,21^. A second source of bias arises because enforcement can act as a displacer of illegal activities, changing resource user behavior and thereby reducing enforcers’ ability to detect occurrences of illegal activity^22,23^. Overcoming these biases requires that detectability analyses explicitly consider the conditionality of violation data (i.e., detection can only occur when there has been a violation), by understanding violation and detection dynamics simultaneously^19^.

Improving the detectability of illegal fishing on the ground needs research that can identify specific factors associated with higher probabilities both of illegal activities and of their detection. This can then support planning of enforcement strategies: how, when, where, and what to focus enforcement on, including what behaviors and by whom^15,16,24^. For example, variability between actors subject to enforcement, or the amount of time invested in specific enforcement activities, can have diverse detectability outcomes^25^. Factors associated with what to target when enforcing include the species being fished and the type of violation^26,27^. For instance, there are some species that, based on their attributes, are more likely to be traded illegally (e.g., totoaba fish in Chinese markets, due to its valuable swim bladder)^28–30^. Various authors have explored how these, and other, factors affect both the incidence of illegal activities and their detectability^17,28,31^.

While studies have explored how detectability varies between species and contexts, most have focused narrowly on illegal fishing activities at sea^15,32^. Studies that assess the detectability of illegal activities by actors more broadly across in fisheries supply chains have been limited. Actors in fish trade supply chains connect fishers with end markets, and therefore can be critical enablers or blockers of illegal activities^33–35^. Moreover, supply chain actors can disproportionately benefit from illegal fishing activities, capturing most of the value that these activities generate^17,36^. Therefore, understanding the involvement of supply chain actors can help improve our understanding of illegal fishing dynamics and better direct enforcement efforts to the most effective intervention points throughout the supply chain.

**Fig. 1:**
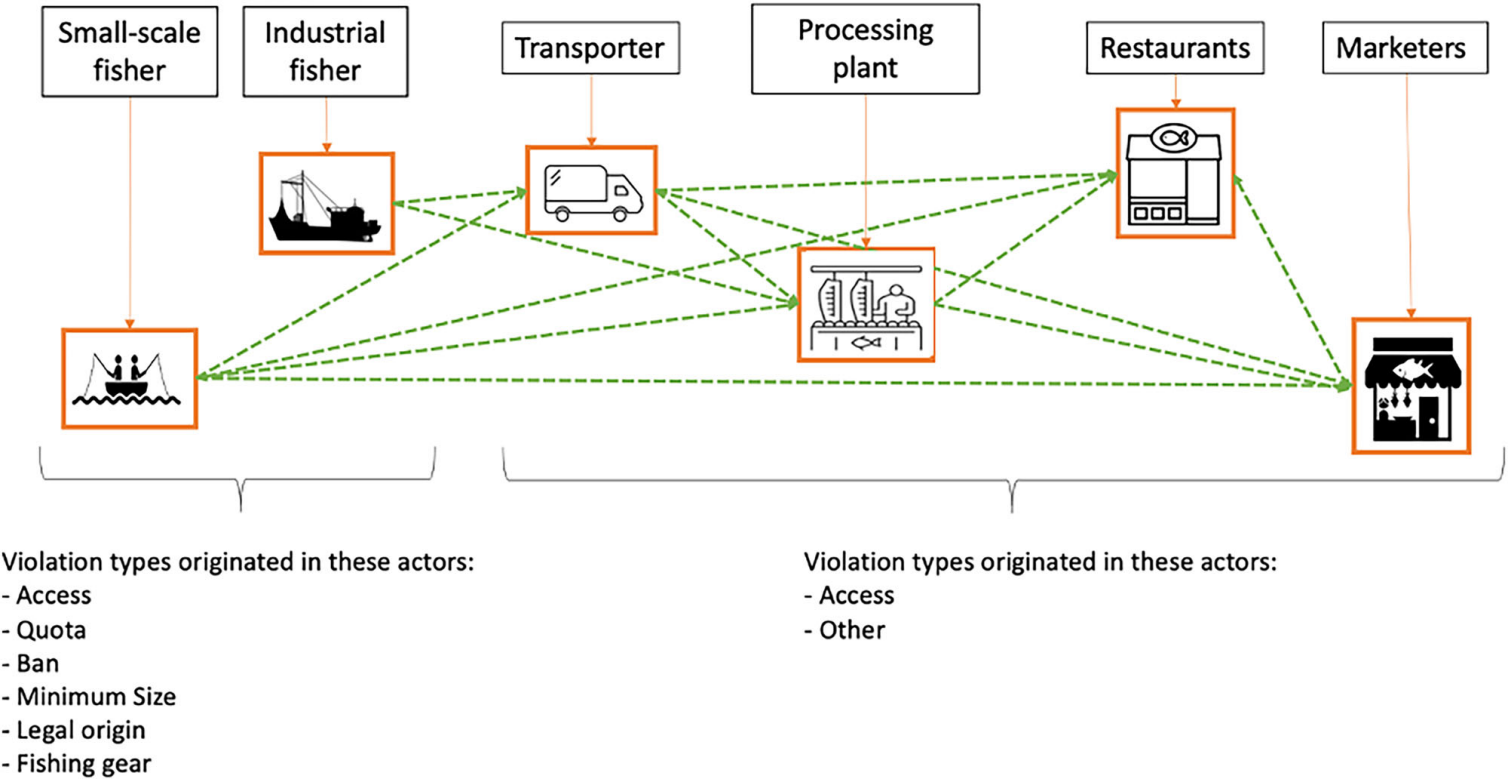
Schematic representation of a fisheries supply chain in Chile, and violation types originated at each step. Green dotted lines represent flow of products between supply chain actors.

Here, we develop a Bayesian Hierarchical Model to assess both illegal fishing activities and their detectability by enforcers. To deal with the conditionality of violations data, we model together the probability of occurrence of violations, and conditioned on their presence, the detectability of those violations, and their predictors. We use a large fisheries enforcement dataset from Chile, which contains information on enforcement efforts (e.g., time enforcing, size of patrolling group) and reports of the violations detected. We focus on illegal fishing activities, defined as those that contravene established national fisheries laws and regulations (and therefore exclude unregulated activities). In the Chilean context, this includes both “illegal” fishing (directly violating regulations) and “unreported” fishing (failing to report or misreporting catches to authorities), as both are explicitly prohibited under Chilean fisheries law. Enforcement patrols included in the dataset are similar in nature (all activities are done on land), and cover all supply chain actors (e.g., fishers, traders, processors, restaurants), all commercial fisheries, and legally binding regulations (most regulations address fishing-level violations, but Chile’s traceability system ensures that illegally harvested products remain detectable as violations throughout the supply chain, as downstream actors cannot obtain valid documentation for products of illegal origin). (Table 1 and Fig. 1). Applying our model, we assess determinants of probability of occurrence of violations (fishery type, violation type) and of detectability (patrolling time, patrol group size, actor type subject to enforcement, and administrative region and year of the patrol), providing relevant management insights to improve enforcement. Our approach of modeling detection and occurrence of violations conditionally and simultaneously can substantially advance understanding of the factors affecting detectability where illegality is present, and clarify how to target enforcement actions across the whole supply chain.

**Table 1:** Predictors for the relative probability of a violation and for detectability

Type of predictor	Predictor	Type/Range
Relative probability of a violation	Species	Categorical: 31 different species or species groups
	Type of violation	Categorical: 7 different types
	Number of enforcers	Continuous: scaled from 0 to 1
	Time enforcing	Continuous: scaled from 0 to 1
Detectability	Actor	Categorical: 8 different actors
	Region	Categorical: 16 different administrative regions

## Results

### Enforcement effort, violations and detectability over time, and model performance

**Fig. 2:**
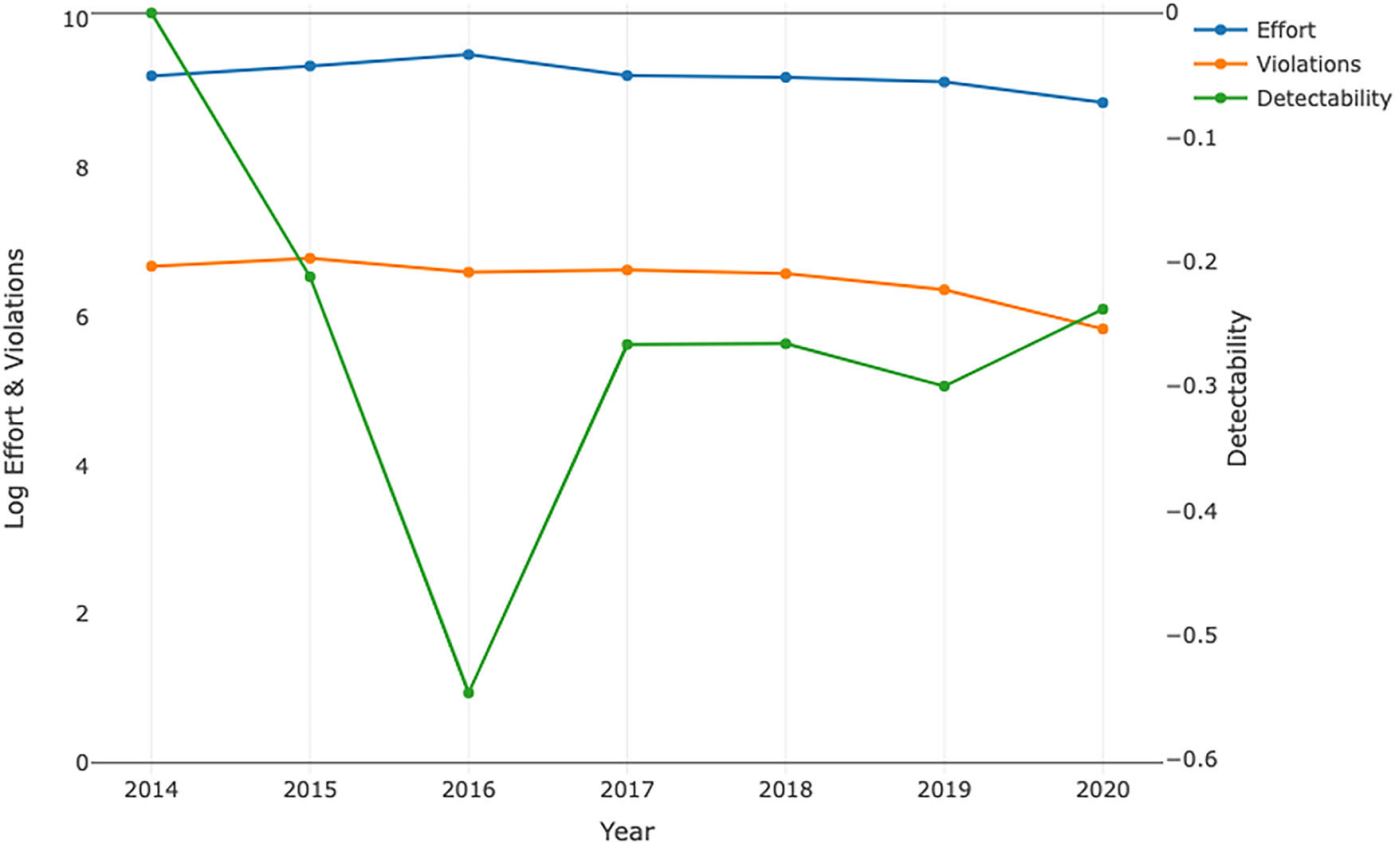
Temporal trends in effort and violations data, and detectability (as estimated by the model). The graph illustrates the changes in enforcement effort (blue), violations (orange), and detectability (green; scale on the right axis) over time. Effort and violations are represented on a logarithmic scale to accommodate the wide range of values observed in the dataset, with values ranging between 0 and 10. Detectability is measured using a separate scale on the right axis due to differences in units.

Total land-based enforcement effort was higher in the first part of the time-period, dropping over time such that effort in 2020 was half that in 2016 (Fig. 2 and Table S1). The number of violations recorded also drops over time, with its minimum value in 2020. Detectability, as estimated by the model, was highest in the first year of our dataset (2014), dropped in 2015 by around 20% and then stayed at roughly the same level (around 75% of the 2014 value) except that it was significantly lower in 2016 (around half of the 2024 value), the year in which effort was highest but violations detected stayed relatively constant. The model had strong predictive power (Table 2). Overall, the model predicts 94% of violations and 99% of non-violations correctly for the Chilean data set, with an F1-score of 0.95 (see “Methods” and Supplementary Material).

**Table 2:** Model performance: data against model predictions

	Dataset		
Model		Not detected	Detected
	Not detected	68737	275
	Detected	169	4581
	Total	68906	4856

The table displays the comparison between dataset values (Dataset, columns) and predicted values obtained from the model (Model, rows), in terms of violations detected and not detected in each enforcement action.

### Detectability and violation probability

Results suggest that, for commercial fisheries in Chile, the mean probability of an enforcement action detecting a violation that has taken place is 0.18 (SD ± 0.14). The mean relative probability of a violation occurring in the context of an enforcement action is 0.34 (SD ± 0.15). The overall probability of observing a violation while on an enforcement patrol, considering both probabilities, is much narrower, with a mean of 0.067 (SD ± 0.10). Therefore, on average, about 7% of enforcement actions lead to violations being detected.

### Predictors of the probability of detecting a violation

**Fig. 3:**
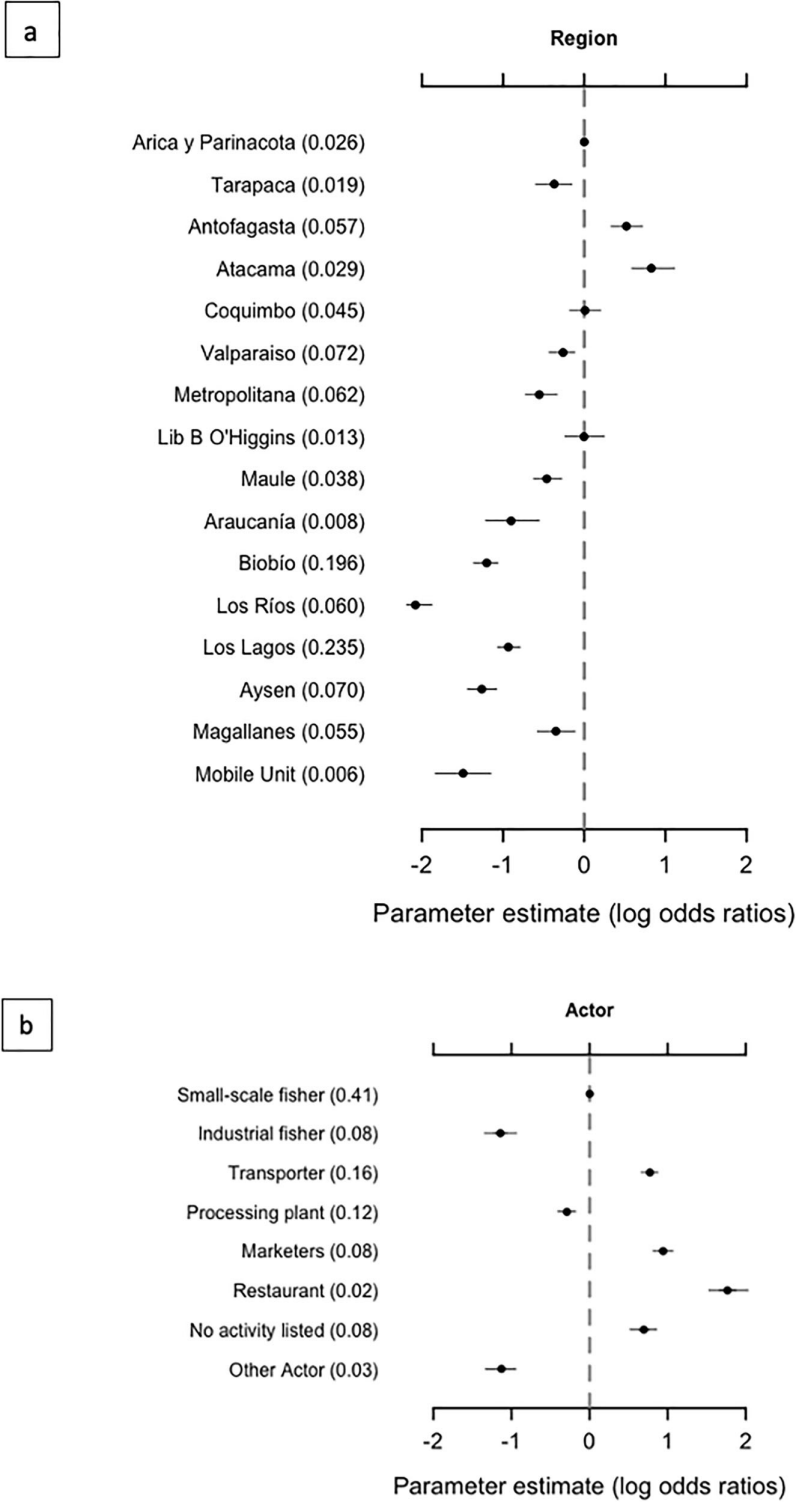
Predictors of the probability of detecting a violation. Mean (dot) and 95% CI (lines) of log odd ratios posteriors for probability of detection of a violation, for **a** each region and **b** actor category, with regards to the reference category (Arica region, and Small-scale fisher, respectively). Values in parenthesis are the proportion of enforcement patrols in each category (the enforcement effort dedicated to that category). Region categories are sorted geographically from north to south (with the mobile unit at the end), and actor is sorted by position in the supply chain (except for no activity listed and other actor).

Our results show that time spent carrying out an enforcement action had a significant and positive effect (mean = 1.79, SD ± 0.14) on the probability of detecting a violation, and the number of enforcers in the group carrying out a particular action has a significantly negative effect (mean = −1.52, SD ± 0.45). The effect of geographical region on the probability of detecting a violation is very heterogeneous, with two regions in the north having high detectability (Antofagasta, Atacama), and Los Rios in the south and the Mobile Unit (which moves between regions) having lower detectability (Fig. 3a). Effort was relatively evenly distributed between regions, except for Biobío and Los Lagos (two of the most important fishing regions in terms of landings). These regions receive 19.6% and 23.5% of the national effort despite having low probability of detection. There was no clear correlation between the proportion of effort dedicated to each region and the probability of detection

Actors towards the end of supply chains (restaurants, marketers, transporters) are associated with a higher probability of detection than resource harvesters (small-scale and industrial fisheries) (Fig. 3b). By contrast, small-scale fishers (with a middling probability of detection) are subject to 41% of the enforcement effort, while restaurants (with the highest probability of detection) receive only 2% of the effort. There is no clear relationship between enforcement effort and actor type.

### Predictors of the relative probability of a violation

**Fig. 4:**
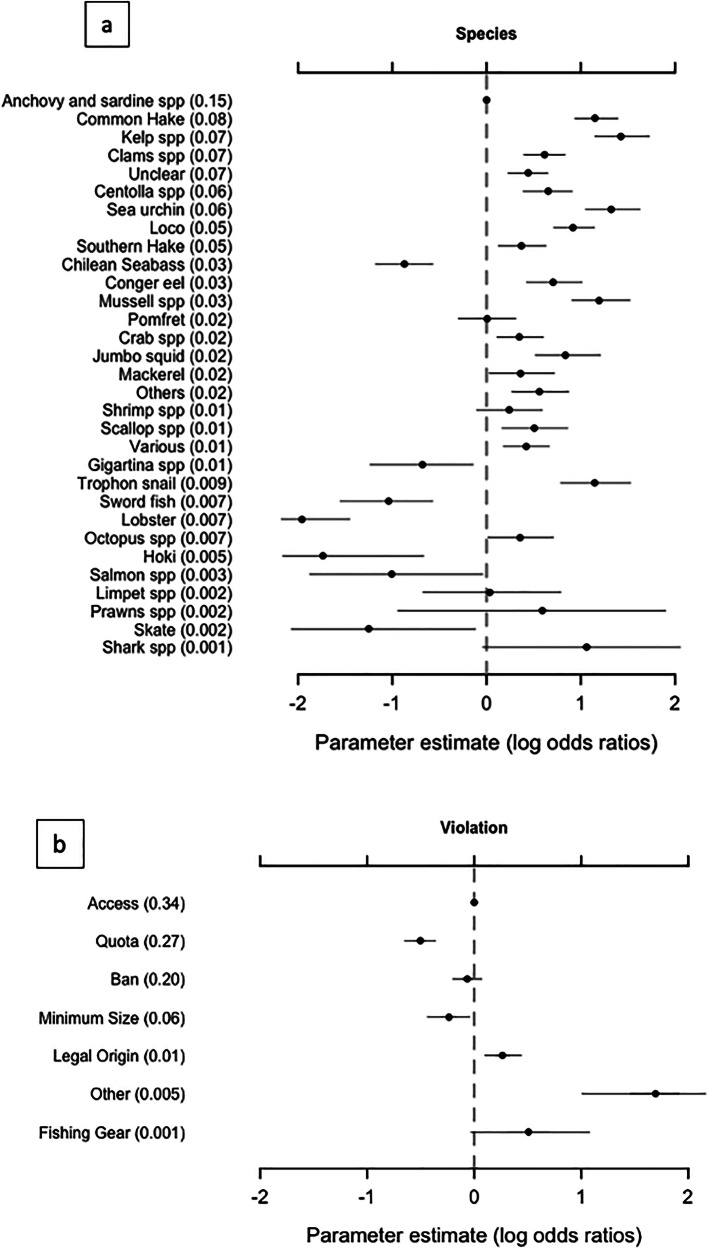
Predictors of the relative probability of a violation. Mean (dot) and 95% CI (lines) of log odd ratios posteriors for the probability of a violation occurring, for **a** species group and **b** violation type, with regards to the reference category (Anchovy, and Access, respectively). Values in parentheses are the proportion of enforcement events in each category (the enforcement effort dedicated to that category). Categories are sorted by enforcement effort.

Enforcement effort varies between species groups, with sardine and anchovy species having the highest proportion of enforcement actions (15%), and common hake the next highest (8%). Several species groups receive <1% of the effort. The effect of species on relative probability of violation is heterogeneous. Many species (including hake and kelp) have far higher relative probabilities of a violation than anchovy species, while the probability for Chilean seabass is significantly lower (Fig. 4a). Species groups receiving less effort are slightly less likely to have a violation, but these estimates are much less reliable due to low sample sizes. With regards to violation type, quotas, bans and minimum size limits have a lower relative probability of a violation than access violations (related to access to fishing permits) (Fig. 4b).

## Discussion

Improving enforcement is crucial to incentivize compliance and reduce the negative impacts of illegal fishing activities^6,37,38^. We present an innovative approach to understanding the capacity of enforcers to detect violations, using a commonly collected but rarely used source of data (i.e., records from enforcement agencies). Our approach splits the modeling approach into two to deal with the conditionality of the process underlying enforcement records (e.g., that detection can only occur when there has been a violation). By doing so, we produced a model with an excellent fit for our case study system (but see below for challenges in separating violation and detection processes in other circumstances). Moreover, the model offers insights into the factors associated with the differences in detectability and probabilities of relative violations, and variation in detectability in space and time. As such, our approach can provide governments and enforcement agencies in our case study (and more broadly) with crucial information about the current efficiency of their efforts, enabling them to assess potential ways to improve future targeting of enforcement efforts to maximize its impact on the underlying rate of violations. While our empirical findings are specific to the Chilean context, the hierarchical Bayesian modeling approach developed here offers a methodological framework that can be adapted to assess detectability and violation probabilities in diverse fisheries contexts globally.

Our results show that, for our Chilean fisheries case study, the overall probability of observing a violation while on an enforcement patrol on land is around 7% (composed of a 34% violation probability and a 18% detectability probability). Few studies have given comparable figures because it is challenging to estimate detectability robustly, having controlled for enforcement effort^21,31^. Studies which have done this tend to involve different contexts, such as wire snares in terrestrial Protected Areas. For example, in experimental trials of snare detection by ranger patrols^31^, found an average snare detectability of 0.2 and^21^ found detectabilities of 0.15–0.26. These studies found that critical factors associated with detectability were related to effort (time spent searching for snares and the number of people in the team). Our results are aligned with these with respect to the length of an enforcement action. It is to be expected that the longer an enforcer searches, the more violations they should find (at least up to a threshold)^13,31^. However, we found a significant negative correlation between detectability and the number of enforcers in a group. This could be due to the increased visibility of large groups of enforcers, allowing more opportunities for violators to perform avoidance strategies and evade detection.

In our case study, we found that actors further up the fisheries supply chains towards end-users (transporters, marketers, restaurants) are associated with an increased probability of detection compared to fishers (whether small-scale or industrial). This could be due to operational characteristics of actors further up in the supply chain, such as spatially explicit operating points (e.g., restaurant compared to moving fishers) and product stockpiling throughout the supply chain, which could facilitate enforcement actions. Moreover, fishers are associated with lower probabilities of detection due to varied reasons such as better detection avoidance skills, ability to move, and more resistance to enforcement activities^10^. The increase in probability of detection which we uncovered, contrasts with the level of enforcement effort targeted at these sectors, with particularly high levels of effort targeting actors with relatively low probability of detection (e.g., small-scale fishers) and very low effort directed at actors with higher probability of detection (e.g., restaurants). While our approach cannot assess causative relationships, reasons for this discrepancy may include the traditional focus of fisheries enforcement on harvesting activities rather than distribution chains, with institutional structures and personnel historically oriented toward monitoring fishers directly, which could lead to higher perceived enforcement risk or ease of operation in familiar contexts. Enforcers may favor targeting fishers because they have established protocols, training, and experience in this domain, making them more comfortable continuing these practices despite potential efficiency gains from shifting focus to other supply chain actors. Our result highlights the importance of increasing the enforcement effort directed at these actors as a way to deter and reduce illegal activities in fisheries. This is relevant for fisheries management in Chile and more broadly. Indeed, there has been increasing recognition by governmental authorities in Chile of the need to expand enforcement activities from fishers to all actors involved in the commercialization of fish^39^. Our results provide solid empirical evidence that this approach is indeed the right path to follow. Moreover, our results are in line with recent calls to consider blue foods as a key component of global food systems, which necessitates consideration of all aspects of sustainability, including supply chains, as products move from production on one side of the world to consumption on another^3,40,41^.

While our analysis focuses on the technical aspects of detection probabilities, we recognize that decisions on enforcement strategies carry significant ethical implications. Shifting enforcement focus across different supply chain actors can have justice, equity, legitimacy and social impact. For instance, small-scale fishers often represent socially and economically vulnerable communities with limited livelihood alternatives^42^, compared to downstream actors like processors and restaurant owners who typically have greater economic resources and alternatives. This disparity should be evaluated and considered when designing enforcement strategies, as it can drive negative social consequences, resistance to regulations and lowered legitimacy of laws^11,43^.

Our results with regard to species groups are in line with previous work which identified species with a higher level of violations (e.g., common hake, loco, kelp spp.)^11^, and we also pinpoint species where there appears to be relatively high probability of violations but no previous evidence (to our knowledge) suggesting illegal activity (e.g., trophon snail, mussels). Moreover, most of the species identified as having high probabilities of violation are also those with some the highest market importance in Chile, while the unexpected findings for others, such as the Trophon snail, demand further investigation into the underlying cause. This inconsistency could also be explained by enforcement effort; species that have not been identified as having relatively high levels of illegal activity in the literature, but we identify here, have been less heavily targeted by enforcers. Moreover, there are intrinsic differences in fisheries and their supply chain that we are not considering in this analysis and can also influence the heterogeneity in the results (e.g., whether products are processed or not, traded fresh or frozen, differences in markup prices along the supply chain, etc). Therefore, combining our results with those from other studies (e.g.^11^, could provide a more complete picture of illegal fishing in Chile, by bringing together modeling and on-the ground approaches to confirm and complement each other.

Overall, our model has the capacity (as shown with our case study) to identify untapped potential for increasing and improving efforts towards actors, species, or types of violations with higher detectability or likelihood of violations. This can provide efficiency gains (i.e., better return on investment) by refocusing enforcement efforts to maximize detectability without necessarily increasing enforcement costs. An example from our case study is restaurants, which had the highest detectability estimate, but where the least effort is currently deployed. This discrepancy is in line with current enforcement strategies in Chile, which do not prioritize restaurants, with the high detectability potentially explained a lack of avoidance strategies used by restaurants due to the low likelihood of receiving enforcement. However, in order to understand the effect of increasing enforcement effort towards a particular target, it is important to consider the dynamism of illegal activities and potential feedbacks, for example through assessing network-based relationships between supply-chain actors, or deterrence or displacement of illegal activity over time^10,22^. The extent to which the deterrent effect of enforcement scales linearly with the perceived or actual probability of being subject to an enforcement activity is likely to vary between actors, locations, and with other factors (e.g., weather, landing activity, past enforcement effort)^21,31^. Additionally, if the overall budget is limited, increases in effort towards one target group will be accompanied by decreased effort elsewhere^8^. Therefore, the overall impact on an agency’s budget of targeting particular groups (and therefore the relative return on investment of refocusing enforcement activities) is also an open question^44^.

Our approach and its application to the case study has provided empirical insights that could guide further study, rather than a blueprint for strategic analysis, especially for other contexts in Latin America and beyond. Annual reassessment and more complex models will be needed to account for the dynamic response of actors to changes in emerging enforcement strategies. This is key since there is a potential interdependence between violation and detection probabilities. Improved detectability can deter potential violators, possibly reducing violation rates as actors adjust their behavior to avoid detection. However, lower violation probabilities may, in turn, decrease detection, influencing the effectiveness of enforcement strategies over time. Building on this, future research should explore dynamic, adaptive management strategies that utilize the strengths of our approach in order to optimize budget allocation adaptively, and which respond in real-time to changes in behavior by updating effort allocation between actors and geographies, as well as considering how changes in detectability might affect violation probabilities and vice-versa. Furthermore, while our current model leverages “inter-annual” data to understand and predict enforcement outcomes, future analysis could integrate “intra-annual” elements. By factoring in seasonality, fishing seasons, and key socio-economic or cultural events throughout the year, the temporal granularity of the model could be improved. This enhancement could advance intra-annual precision of enforcement activities, potentially elevating detectability and allowing for the anticipation and strategic allocation of enforcement resources during peak periods of illegal activity. Finally, a comprehensive economic analysis of enforcement impacts on supply chain actors or different enforcement strategies was not possible with our dataset. Such analysis would require data on fines and/or seizures, as well as enforcement costs (personnel, transport, equipment), which were not available for this study. Future research integrating such data could provide valuable insights into how enforcement strategies economically affect different supply chain actors, how these impacts might influence compliance behaviors, and their return on investment.

Despite these caveats, our approach can help advance understanding of illegality in fisheries in particular and other wildlife trade contexts more broadly. Firstly, by providing a replicable approach to analyzing data from enforcement and violations records. Indeed, our approach considers factors associated with differences in detectability but also accounts for the conditionality of the data related to enforcement actions. Using a Bayesian hierarchical approach with conditional logit functions, we can accommodate these data types more efficiently and provide insights into the factors associated with the modeling processes, considering the relative probability of a violation and detectability together. While we tested the model with a configuration in which factors are unambiguously associated with one category or the other, this might not work everywhere; more research is needed to determine and test which factors are associated with detectability, probability of violations, or both in different circumstances. Secondly, we propose a shift in attention from harvesters to the whole supply chain when dealing with illegality in fisheries and potentially wildlife markets in general. This is not without its challenges. Enforcement agents would require new sets of skills and training, while the risks associated with enforcing rules vary with actor type. Different actors along the supply chain may have different avoidance strategies^10^ or use different levels of violence to prevent enforcement. Researchers would also need to broaden their methodological approaches to account for actors and sectors that are usually not on their radar, potentially including international criminal gangs^45^.

Research on detecting illegal activities has advanced strongly in recent years, through the use of new technologies, for instance, satellite imagery (e.g., Global Fishing Watch^32^). However, these technologies rarely cover small-scale fisheries, which make up more than half of global catch and employ the vast majority of fishers, but are usually neglected in illegal fishing research^46,47^. Analyses like ours, which use datasets collected regularly by enforcement agencies on the ground but are rarely used in detectability research, can incentivize better data collection protocols. Expanding the extent of data collection and analyses can provide a much broader picture of illegal fishing activities, resulting in positive feedback in which better data are collected, which feeds better models, better informing effective management actions aimed at reducing the extent of illegal activities^19^. For these data to effectively inform management, however, appropriate collection protocols need to be in place to control biases. For instance, increased spatial evenness of data collection can produce data that better represent the true extent of illegal activities^22^ but this trades off with focusing effort in areas with higher probability of detection and higher violation rates.

While we have focused exclusively on the capacity of the model to explain the factors that affect the probability of a violation and its detection, our approach could be used prospectively by using the posterior distribution results to assess the effect of different hypothetical effort strategies on detectability, and validating a model trained on one part of the dataset using the other part of the dataset as test data^44^. This is a key step for turning this approach into a predictive tool that can better help reduce illegal activities, which we left for future research due to current data availability limitations. In applying this approach, however, there is a need to reduce implementation barriers. User-friendly apps, such as R Shiny (interactive app from R analyses), can be used to bypass the need for Bayesian modeling capacities at enforcement agencies, but strong quantitative skills will still be needed for monitoring indicators (e.g., changes in detectability) institutionally to improve the application of the analyses. Moreover, enforcement agencies (e.g., SERNAPESCA) could implement empirical tests of how shifts in enforcement effort affect the distribution of violations and the ability to detect such violations (e.g., using $\Delta$CPUE-$\Delta$E plots^13^,). This can further help to understand the functional form of relationships between, for instance, deterrence and enforcement activity in a quasi-experimental setting, as well as assessing responses in dynamic socio-ecological systems. Finally, field trials would be informative on how to best deploy limited budget in a dynamic socio-ecological system, acknowledging feedbacks and trade-offs within strategies, forming the basis for an adaptive management framework.

Fisheries are a crucial source of food and livelihood for millions around the world^1–3^. Improving enforcement can help reduce illegal fishing activities and advance the sustainability of fisheries^17^. Our approach presents a novel way to assess the capacity of enforcement to detect violations, and our results show the need to expand these types of analyses to include not only fishers but all actors involved in fisheries supply chains. Improving detectability is an especially effective way to reduce illegal fishing activities in contexts where enforcement agencies suffer from low budgets, limited capacities, and where technological advances might not be available^46,47^. However, enforcement would not solve all problems around illegal fishing^27,48^. It needs to be complemented with a better understanding of the diverse motivations (such as instrumental, normative, or legitimacy-based) that drive people to engage in illegal activities^9,49–51^, and of the trade-offs facing enforcement agencies in complex and dynamic systems with multiple interacting supply chains. Advancing the sustainability of fisheries requires improving the capacity of enforcement agencies to detect illegal activities efficiently and effectively, complementing other efforts that aim to promote compliance in the long term.

## Methods

### Case study: Chile

Chile is one of the most important fishing nations in the world, with a commercial sector contributing an average of 2.6 million tons a year and directly employing more than 100,000 people^39^. Chile’s coast is highly productive due to the cold, nutrient-rich Humboldt Current. Because the country’s coastline spans more than 4000 km on the north-south axis, there are a great diversity of climates, fish species, and fisheries ranging from deep-water industrial bottom-trawlers to coastal inshore gatherers^52^. Commercial fisheries provide an essential economic activity, feeding a large domestic market, concentrated at a central fishing terminal, and a key export sector^27^. While fisheries are largely regulated in Chile, with science-informed quotas, territorial user rights, and a fishers register, illegal activities are widespread^26,53^. For some species, illegal landings are estimated to be larger than legal ones^5,36^. SERNAPESCA is Chile’s central fisheries enforcement agency, and reports on both its enforcement efforts and violations. This agency has over 900 staff, 46 offices, and an annual budget of ~40 million USD.

### Data

We used a dataset containing reports of each enforcement activity performed in 2014–2020 by SERNAPESCA for commercial wild-caught fisheries across the country (*n* = 77,820). We obtained these anonymized data via a data transparency request to SERNAPESCA, a legal mechanism in Chile that is publicly available. Patrol activities by SERNAPESCA are all carried out on land and do not include activities at sea. The same enforcers target different actors throughout the supply chain, and are similarly equipped for any of the actors they are targeting. As such, enforcement activities are similar in nature, so factors that explain differences in detectability can be assessed. It is important to note that Chile’s fisheries law establishes a mandatory traceability system that creates a direct link between illegal fishing and downstream illegality. This system requires documentation to accompany fish products throughout the supply chain that must be presented during inspections. Similarly, this system allows to differentiate domestic and imported products (which are overseen by customs authorities). When fish are caught illegally (e.g., during closed seasons, exceeding quotas, or without proper licenses), no legal documentation can be issued. Consequently, any downstream actor in possession of these products is automatically in violation because they cannot provide the required traceability documentation. This mechanism ensures that the illegal status of products persists throughout the supply chain.

In the dataset, each enforcement activity has information on: date; time spent enforcing; administrative region where the enforcement activity occurred; the number of enforcers that participated; and the species, type of violation, the actor targeted by a specific enforcement activity and whether a violation was found or not (Table S2). For species, actor, and type of violation, we considered all the categories established by SERNAPESCA (Table S3). For the type of violations, the category “Other” included an ensemble of minority class violations: activities in restricted areas such as Marine Parks, Marine Reserves, or restricted zones, and operations within the first nautical mile. Other violations involve unauthorized landing points, shark finning, and obstruction of enforcement efforts. Additionally, they include issues with statistical reporting, such as failure to provide or falsification of information.

We categorized predictors into two categories: those that predict the relative probability of a violation and those that predict the detectability by enforcers of the violation (Table 1). This can be challenging, because in reality predictors have complex interacting effects on violations and detectability^8^. However, given data limitations we were only able to fit models in which a predictor was assigned to one or another category. In our model, species (i.e., fishery) and type of violation were categorized in the violation predictors category because they directly affect what to target when enforcing and the likelihood of an illegal fishing activity. For instance, some species have attributes that makes them more attractive as targets of illegal activities (e.g., totoaba fish in Chinese markets, due to its valuable swim bladder)^28–30^; and some types of violations are much more profitable than others (e.g., fishing over the quota rather than fishing during reproductive ban when there is no market)^11^.

In our model, predictors associated with detectability relate to how, when, where and on whom to target enforcement. “How” predictors relate to the number of enforcers that participate in an enforcement patrolling action (mean = 2.0; SD = 1.1), and the time they spent enforcing (in minutes) (mean = 311.6; SD = 217.9). “When” and “where” predictors are year in which the patrolling action took place, and region (Chile’s administrative region where the enforcement action took place), to control for geographical and long-term temporal heterogeneity. Finally, the “who” predictor was the type of actor at whom the enforcement is targeted (e.g., fisher, restaurateur, transporter). With regard to the time predictor, we assume deterrence is instantaneous and therefore any effect of enforcement happens in the same time-period as the enforcement action. We assume this because of data constraints: each specific enforcement action is targeted to a specific combination of actor, violation, and species, and is bound to an outcome at the same time-period, so we could not assume a more realistic lagged effect of enforcement. For all predictors, from the raw data, we collapsed similar categories (e.g., adding small-scale boats to small-scale fishers) to reduce the number of predictor categories (Table 1). Moreover, when, in some cases, there was more than one actor, type of violation, or actor as the target, we used the initial target objective of the action as our predictor.

### Model

Our Bayesian Hierarchical Model addresses the fundamental challenge in analyzing enforcement data: detectability is conditional on the presence of a violation. This conditionality creates a two-stage process that is challenging to assess by standard statistical approaches. To address this, our model explicitly represents this conditional relationship through two linked logistic functions. The first function was used to predict the relative probability of a violation (${{logit}(v}_{i})$). The second function was used to predict the detectability if there was a violation (${{logit}(p}_{i})$). This structure allows us to separate these two processes and identify which factors influence each component. Without this hierarchical structure, we would conflate factors affecting violation rates with those affecting detection capabilities, potentially leading to misleading conclusions about enforcement effectiveness. The Bayesian implementation offers several advantages over frequentist approaches for this application: it naturally accommodates the hierarchical structure through probabilistic conditioning, it provides full posterior distributions rather than point estimates, allowing us to better quantify uncertainty in our estimates and it allows for more flexible incorporation of prior information when available.

We implemented the conditionality by drawing an outcome from a Bernoulli distribution with its probability parameterized by the violation logit function (${s}_{i}$). We then multiply this outcome (1 or 0) by the probability obtained in the second logit function representing detection (${P}_{i}$). The product was a probability that we used as the *p* parameter of another Bernoulli distribution (${y}_{i}$) to model the response variable from the data of the presence or absence of a violation (see below):

1 $${y}_{i}\, \sim {Bernoulli}({P}_{i})$$

2 $${P}_{i}\,={p}_{i}{s}_{i}$$

3 $${s}_{i}\, \sim {Bernoulli}({v}_{i})$$

4 $${{logit}(p}_{i})=\,{\mu }_{p}+\,{b}_{1}{T}_{i}+\,{b}_{2}{N}_{i}+\,{b}_{3}\left[{R}_{\left[i\right]}\right]+\,{b}_{4}{[A}_{[i]}]\,+\,{b}_{5}{[Y}_{[i]}]$$

5 $${{logit}(v}_{i})=\,{\mu }_{v}+\,{b}_{6}{[V}_{[i]}]+\,{b}_{7}{[S}_{[i]}]$$

Where ${y}_{i}$ is the Bernoulli response variable (i.e., 1 or 0 representing presence or absence of a violation) for each enforcement activity $i$; ${\mu }_{p}$ and ${\mu }_{v}$ are the intercepts for the logit functions; ${b}_{1\ldots 7}$ are the parameter estimates: ${b}_{\mathrm{1,2}}$ are single estimates for each continuous predictor (${T}_{i}$ is time enforcing and ${N}_{i}$ is the number of enforcers), and ${b}_{3\ldots 7}$ are estimates for each category of the categorical variables (${[R}_{[i]}]$ is region, ${[A}_{[i]}]$ is actor, ${[Y}_{[i]}]$ is year, ${[V}_{[i]}]$ is the type of violation, and ${[S}_{[i]}]$ is species). We used uninformative uniform distribution priors ${b}_{1\ldots 7} \sim {Unif}(-\mathrm{2.2,2.2})$ for the intercepts and the continuous and categorical variable coefficients. These represent the log odds ratio of the difference in the probability of each category (in this case, we set the uninformative range to be −90 to 90%) to a reference category which was set to zero for each variable. The range of −90% to 90% for the uniform distribution priors was chosen to balance a non-informative prior, which avoids introducing bias, with practical constraints on the prior space, thus reducing the computational need of the model. This range is broad enough to capture a wide array of possible outcomes but prevents the model from exploring an excessively large parameter space.

We tried various model specifications, starting with a null model with no predictors. We then added length of the enforcement visit (time in hours), year, the number of enforcers present in the visit (group size) and the categorical predictors (species, type of actor and violation, and region). Our model then accounts for the variability of different regulatory frameworks and rules by including type of violation as a predictor of violation probability, allowing us to estimate species-specific violation probabilities while controlling for the regulation that was being enforced. This approach captures the composite effect of each species’ regulatory framework on the likelihood of illegal activity. Moreover, we tested the use of polynomials to account for potential non-linearities in the continuous variables (time and group size) and an interaction term. Finally, we also tested some alternative hypothesis for whether the spatial (administrative region) and temporal (year) predictors were related to detection or violation. To ensure the robustness of our model, we checked for collinearity among all predictors and excluded those with high collinearity (*r* > 0.7). After several iterations, the best fit model (with the lowest wAIC, see Table S4) included the continuous variables time and group size (scaled from 0 to 1), and actor, region, and year as predictors of detectability. Species and type of violation were included as predictors of the presence of a violation. We tested the final model convergence with Rhat, a proxy for how well chains (*n* = 3) mix. With 3000 iterations (600 for warmup and 2400 for sampling), all Rhat values for model posterior parameters were <1.1, which is considered appropriate convergence^54^. We then performed model fit verification via posterior checks, but varied the threshold to determine if each activity was assigned as a violation detected or not detected (0 or 1). The “normal” threshold would be 0.5, but we varied this to see if the predictive capacity of the model could increase, assessed with the F1 score. F1 is a score that combines precision (correct positive predictions relative to total positive predictions) and recall (correct positive predictions relative to total actual positives) and is recommended for unbalanced samples where the aim is to improve prediction on the positive minority class. The threshold that provided the highest F1 score and, therefore, the better fit was 0.24 (Fig. S1), with an F1 value of 0.95. We use R Studio to run the model.

## Supplementary information

Supplementary information

## Data Availability

The data are available at: https://github.com/rodgpt/Detectability-of-illegal-activities.
