# Supplementary material for: Improving detectability of illegal fishing activities across supply chains
Source: NPJ Ocean Sustain. 2025 Jun 21;4(1):34. doi: 10.1038/s44183-025-00134-5 (PMC12225464; doi:10.1038/s44183-025-00134-5)
Supplement: Supplementary file 1 — Supplementary information [file 44183_2025_134_MOESM1_ESM.docx]

**Supporting Information**

**Table S1.** Number of effort actions and violations per year**.**

| **Year** | **Effort** | **Violations** |
| --- | --- | --- |
| 2014 | 73284 | 1957 |
| 2015 | 84783 | 2338 |
| 2016 | 91541 | 1561 |
| 2017 | 75975 | 1583 |
| 2018 | 76944 | 1446 |
| 2019 | 57508 | 1326 |
| 2020 | 43772 | 877 |

**Table S2**. Number of effort actions and violations found for each actor, violation type, region and species.

| **Category** | **Type** | **Effort** | **Violations** |
| --- | --- | --- | --- |
| **Actor** | Small-scale fisher | 30827 | 3399 |
|  | Industrial fisher | 6140 | 125 |
|  | Marketers | 5834 | 1575 |
|  | No activity listed | 5733 | 207 |
|  | Other Actor | 2368 | 599 |
|  | Processing plants | 9284 | 802 |
|  | Restaurant | 1679 | 633 |
|  | Transporter | 11753 | 2962 |
| **Violation** | Access | 25070 | 3219 |
|  | Ban | 15132 | 2544 |
|  | Fishing Gear | 615 | 89 |
|  | Legal Origin | 7265 | 2162 |
|  | Min Size | 4962 | 534 |
|  | Quota | 20237 | 1680 |
|  | Other | 337 | 74 |
| **Region** | Arica y Parinacota | 1954 | 175 |
|  | Tarapaca | 1430 | 213 |
|  | Antofagasta | 4202 | 1503 |
|  | Atacama | 2190 | 796 |
|  | Coquimbo | 3352 | 781 |
|  | Valparaiso | 5304 | 1128 |
|  | Metropolitana | 4566 | 1243 |
|  | Lib B O'Higgins | 1008 | 245 |
|  | Maule | 2838 | 546 |
|  | Araucanía | 660 | 173 |
|  | Biobío | 14483 | 900 |
|  | Los Ríos | 4424 | 137 |
|  | Los Lagos | 17356 | 1728 |
|  | Aysen | 5192 | 285 |
|  | Magallanes | 4073 | 345 |
|  | Mobile Unit | 488 | 90 |
| **Species** | Anchovy and sardine spp | 11441 | 309 |
|  | Centolla spp | 4908 | 504 |
|  | Chilean Seabass | 2242 | 102 |
|  | Clams spp | 5642 | 972 |
|  | Common Hake | 6429 | 1275 |
|  | Conger eel | 2756 | 530 |
|  | Crab spp | 2142 | 261 |
|  | Gigartina spp | 722 | 21 |
|  | Hoki | 346 | 0 |
|  | Jumbo squid) | 1307 | 170 |
|  | Kelp spp | 5285 | 1865 |
|  | Limpet spp | 133 | 14 |
|  | Lobster | 499 | 4 |
|  | Loco | 4092 | 781 |
|  | Mackerel | 1470 | 127 |
|  | Mussell spp | 1998 | 398 |
|  | Octopus spp | 503 | 55 |
|  | Others | 1802 | 216 |
|  | Pomfret | 1801 | 123 |
|  | Prawns spp | 162 | 5 |
|  | Salmon spp | 238 | 14 |
|  | Scallop spp | 810 | 175 |
|  | Sea urchin | 4533 | 549 |
|  | Shark spp | 81 | 14 |
|  | Shrimp spp | 835 | 147 |
|  | Skate | 216 | 4 |
|  | Southern Hake | 3801 | 302 |
|  | Sword fish | 559 | 37 |
|  | Trophon snail | 674 | 201 |
|  | Unclear | 5032 | 828 |
|  | Various | 1159 | 299 |

**Table S3**. Description of action and violation category types, as defined by SERNAPESCA.

| **Category** | **Type** | **Description** |
| --- | --- | --- |
| **Actor** | Small-scale fisher | Artisanal fishers (boats <18 meters in length), divers and shore gatherers |
|  | Industrial fisher | Industrial boats (>18 meters in length) |
|  | Marketers | Actors who commercialize marine products |
|  | No activity listed | Actors not registered/ no actors found |
|  | Other Actor | Ensamble of minority class actors |
|  | Processing Plants | Actors involved in processing marine products |
|  | Restaurant | Actors selling marine products in restaurants |
|  | Transporter | Actors that transport marine products |
| **Violation** | Access | Lack of permits to operate in the fisheries sector, including supply chain |
|  | Ban | Fishing or commercializing products during a reproductive ban |
|  | Fishing Gear | Fishing with prohibited gears |
|  | Legal Origin | Lack of documentation regarding origin of fishing products |
|  | Min Size | Fish products below permitted size |
|  | Quota | Fish products that have been extracted above regulated quotas |
|  | Other | Ensamble of minority class violations |

**Table S4.** Comparison of model performance with wAIC.

| Model | Detectability Predictors | Violations Predictors | wAIC |
| --- | --- | --- | --- |
| Null model | NA | NA | 24920 |
| *Model 1* | *time, group size, region, actor, year* | *species, violation* | *21472* |
| Model 2 | time, group size, region, actor | species, violation, year | 22174 |
| Model 3 | time, group size, actor | species, violation, year, region | 22552 |
| Model 4 | time, group size, actor, year | species, violation, region | 22684 |
| Model 5 | time, group size, region, actor, year time*group size | species, violation | 22161 |
| Model 1 with 2nd order Polynomials | time, group size, region, actor, year | species, violation | 23690 |
| Model 1 with 3rd order Polynomials | time, group size, region, actor, year | species, violation | 23161 |


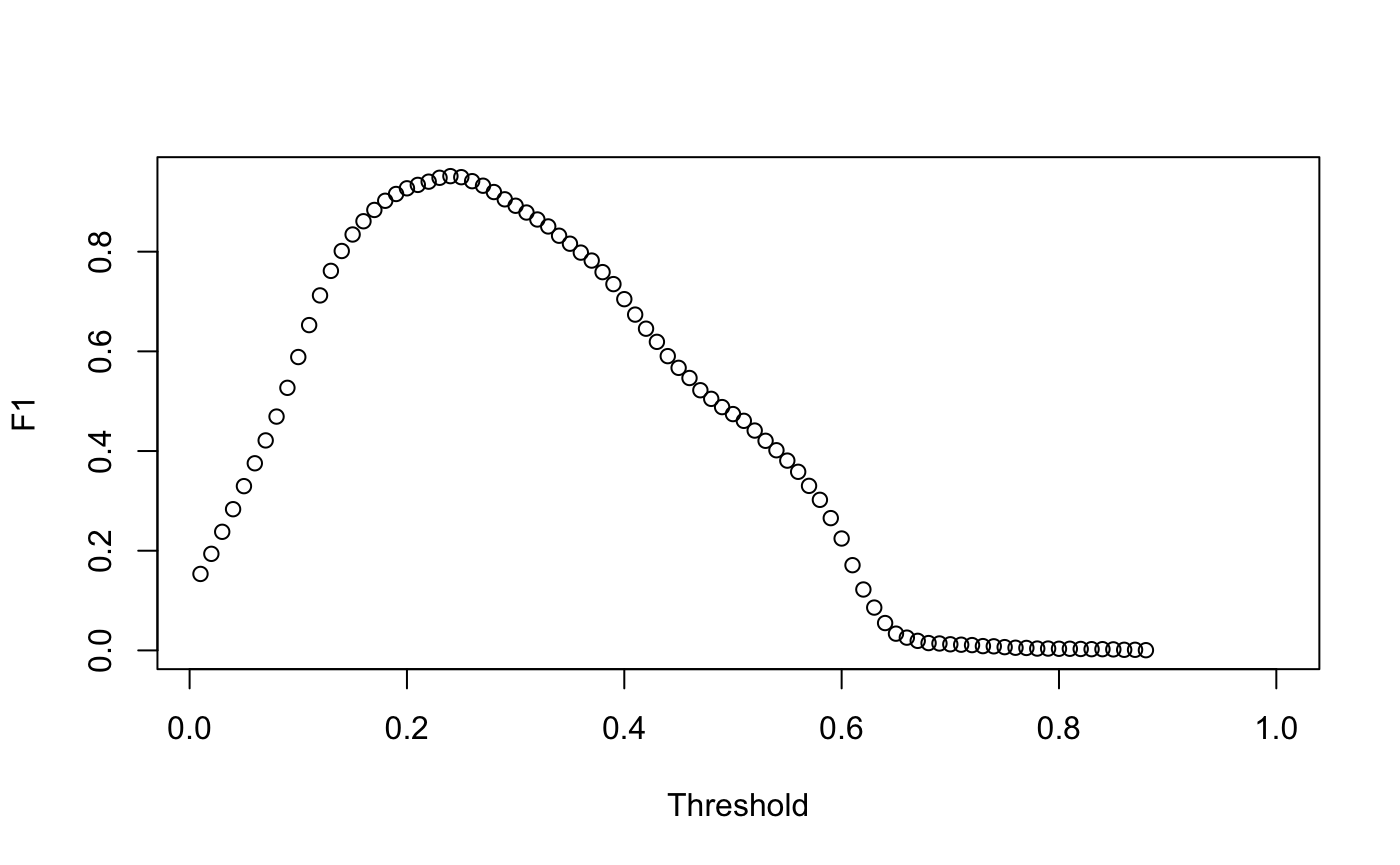
**Figure S1.** Analysis of F1 scores for different thresholds to determine if the probability was assigned as a violation detected or not detected.
